# Impact of an implicit social skills training group in children with autism spectrum disorder without intellectual disability: A before-and-after study

**DOI:** 10.1371/journal.pone.0181159

**Published:** 2017-07-17

**Authors:** Jokthan Guivarch, Veena Murdymootoo, Sara-Nora Elissalde, Xavier Salle-Collemiche, Sophie Tardieu, Elisabeth Jouve, François Poinso

**Affiliations:** 1 Department of Child Psychiatry, APHM, Marseille, France; 2 Faculty of Medicine of Marseille, Aix-Marseille University, Marseille, France; 3 Department of Medical Evaluation and Public Health, APHM, Marseille, France; TNO, NETHERLANDS

## Abstract

**Introduction:**

Children with Autism Spectrum Disorders (ASDs) have problems with social skills. Social skills training groups are among the proposed therapeutic strategies, but their efficacy still needs to be evaluated.

**Objective:**

To evaluate the efficacy of an implicit social skills training group in children with ASDs without intellectual disability.

**Methods:**

A before-and-after study of children with ASD without intellectual disability was conducted in a child psychiatry day hospital, where they participated in an implicit group with cooperative games. Their social skills were assessed using the Social-Emotional Profile (SEP), the Childhood Autism Rating Scale (CARS), and the empathy quotient (EQ) before and after 22 weeks.

**Results:**

Six patients aged 9 to 10 years old were evaluated. A significant increase in overall adaptation and social skills (median 8 and 7.7 points) in the SEP was demonstrated in addition to a significant reduction in the CARS score (median: 4 points), including in the field of social relationships. The EQ increased two-fold.

**Discussion—Conclusion:**

This implicit group improved the children’s social skills. It would be interesting to evaluate the maintenance of these skills over time, examine more widespread results, and compare implicit and explicit groups.

## Introduction

Autism spectrum disorders (ASDs) are serious disorders that occur in early childhood and affect development [1,2].

In the American Psychiatric Association’s DSM5 classification [2], ASDs have different specifications, depending on the severity of the condition; whether the disorder is associated with a medical condition; and whether there is an intellectual disability, altered language or even catatonia [2].

Epidemiologically, in a 2011 meta-analysis, Fombonne found the prevalence of autism to be approximately 20 to 30/10,000, or 1/400, and the prevalence of all ASDs to be 90 to 120/10,000 [1], or 1/100 (1%), which is included in the DSM5 classification [2]. The majority of subjects with ASD have comorbidities [3,4], the most common being an intellectual disability (30% of autistic children have moderate retardation, and 40% have profound retardation) [5,6], ADHD [7], epilepsy [5], and anxiety disorders [2,6]. The term “ASD without intellectual disability (ID)” or “ASD without mental retardation” includes high-functioning autism and Asperger’s syndrome, a diagnostic entity that is not separated in the DSM5 due to the absence of a true criterion to distinguish Asperger’s syndrome as its own diagnostic category [8]. Some research is currently focused on the subgroup of ASD without ID [8–10], which is expected to have a better prognosis than those children with comorbid intellectual disabilities [2,6]. However, these subjects also present with notable difficulties in their social skills, manifested by trouble with interactions and communication [8,10–12]. These difficulties affect their autonomy and quality of life [13,14] and increase their social anxiety, which then contributes to worsening social deficiencies and isolation [15] and explain why care is also necessary [8,11]. Of the therapeutic strategies proposed for this population, social skills training groups [11] are treatments recommended by the French National Authority for Health (Haute Autorité de Santé, HAS) (2010 Report on Autism and Other Pervasive Developmental Disorders [6]]. The literature suggests social skills training groups, which are generally based on a predominantly explicit mode [8–12], meaning that they use explicit learning along with the teaching of theory and then practice [16,17].

Here, we present the results of an original intervention in children aged 9 to 10 years old with ASD without ID. The originality of our intervention lies in the fact that we have developed a social skills training group that uses cooperative games based on an implicit modality. Thus, the group relies on learning from ecological situations without background theoretical teaching.

## 1. Social cognition and social skills training groups in children with ASD

### 1.1. Social cognition in autism

Social cognition is defined as the set of mental processes involved in social interaction [18]. It is altered in people with ASD [10] due to a deficit of theory of mind [19,20]; dysexecutive syndrome [21–23], which more specifically affects planning [21], mental flexibility [24], inhibition [21], and attentional control [25]; and weak central coherence [26]. These deficits may be combined with perceptual overfunctioning [27], resulting in local and sequential processing of information, increased memorization of detail, and difficulty in conducting an overall analysis of information [28].

Empathy is a component of social cognition [29]. It is defined as “the drive or ability to identify emotion and thoughts in another person and to respond with an appropriate emotion” [29–31]. Empathy has two components: an affective component, which is the appropriate emotional response to the affective state of another person, and a cognitive component that includes understanding the mental states of others, which implies that putting oneself in someone else’s place involves an efficient theory of mind as a prerequisite [30,32–34]. People with ASD experience difficulty showing empathy, given the lack of theory of mind, and difficulty reading emotions, which is inherent to their disorders [30,31] (see above).

First targeting adults with ASD without ID, Baron Cohen developed three questionnaires: the Autism Spectrum Quotient, the empathy quotient (EQ), and the systemizing quotient (SQ) [29]. Adults with ASD without intellectual disabilities have significantly lower empathy scores than people without this disorder. The empathy quotient is inversely correlated with the autism quotient, implying that there are more marked autistic traits for a more altered empathy quotient; according to Cohen, this relationship makes ASD “an empathy disease” [30,31]. From the EQ and SQ scores, Goldenberg identified “5 brain types” [35]. Autistic people without ID are said to be type S or extreme S (S for systemizing)—meaning low or very low empathy abilities—in line with the Extreme Male Brain theory, which was developed by Baron Cohen [29,31,35,36]. According to this theory, the cognitive profile observed in ASDs is an extreme of the normal male profile, “in whom systemizing is significantly better than empathizing” [36].

### 1.2. Social skills

Social skills are defined by Trower, Bryant, and Argyle as the set of abilities that aid in understanding messages and expressions communicated by others and responding in a socially appropriate manner with verbal and nonverbal behaviors that could influence the environment sufficiently to achieve one’s personal objectives [37]. Nonverbal behaviors, which are normally synchronized with words, include gestures, mimics, gazes, and postures. Sociopragmatic aspects can be described as conversational social skills. They include pragmatic verbal (prosody, rhythm, metaphor, understanding implicit meaning, particularly irony, etc.) and nonverbal elements [8]. These sociopragmatic aspects encourage the subject to take turns when speaking and allow the subject to engage in or end a conversation appropriately [38].

Typically, in normotypic subjects, social skills are acquired unconsciously. The need for learning is necessary only if the subject is confronted with “an unusual or difficult situation” and requires new responses [39].

People with ASD are at a particular disadvantage because they lack or do not yet have certain prerequisites for social skills—such as imitation, joint attention, and the reading of emotions—because of their central coherence deficiency, the deficit in theory of mind, and the dysexecutive syndrome mentioned above [8,10,40]. Therefore, they have trouble identifying emotions, categorizing facial expressions, and prioritizing information [8]. The pragmatics of the language is altered [8,41] on a verbal level, with problems affecting prosody, flow, sound level, speech (with long, richly detailed monologs most often about restricted interests), and odd verbal expressions that give a “pedantic” or “sententious” character to the subject’s speech. Nonverbal communication is also disrupted with an unusual gaze, reduced facial expressions, and spontaneous gestures [8]. These alterations necessitate extensive learning of social skills, in the form of social skills training [10,40].

### 1.3. Learning and social skills training groups

There are two ways of learning: explicit and implicit [16,17].

Explicit learning (or “by instruction”) is learning that is instituted explicitly with teaching, rules, and methods through steps aimed at acquiring knowledge. It often requires costly efforts in terms of concentration and motivation [17].

Implicit learning is primarily used when exploring the environment, social activities, and games. From a cognitive perspective, implicit learning is not costly [17]. This type of learning is “the ability to learn without thinking about it.” We would be “sensitive to the regularities of the surrounding world” and learn from those regularities [16]. This “learning without knowing it” involves “an adapted change in behavior following a repeated confrontation with a structured situation” that does not involve conscious attention processes [42]. Implicit learning is said to be independent in terms of intelligence [43] and is maintained over time [44]. It would allow for transfer, so the learning could be transposed to a new situation—also known as generalization—if there were commonalities between the original situation and the new situation [45]. A recent meta-analysis shows that implicit learning is not deficient in autistic subjects [46].

Social skills training is a structured method derived from cognitive and behavioral interventions [8,47] and social learning theories [8], and it “aims to teach the social skills that are necessary in interpersonal relationships and to promote the maintenance and generalization of these skills in the patient’s real life” [38]. It is recommended by the HAS in ASDs [6]. Such training is quickly becoming available in the form of structured groups, which are known as social skills training groups [8,11,12,38,48–50].

These groups are designed to make the patient socially competent in interaction situations so that he or she can participate in social life with age-based objectives through group exercises [39]. In a 2013 Cochrane review, Reichow, Steiner and Volkmar showed that there is an improvement in social skills in these groups and a decrease in feelings of isolation, which is consistent with a better quality of life. [11]. For these groups to be efficient, the participants must have a good level of formal language usage and no behavioral problems [39]. There should be homogeneity in the group with regard to the participants [39], both verbally and cognitively [51]. A small number of participants is preferable, between 4 and 8 according to different authors [8,38]. Finally, patient and parent adherence is also required [39]. The groups meet on a weekly or biweekly basis [8,38] and are typically led by two trained therapists [38,39]. The sessions are structured and predictable [51] with a “standard sequence of activities” [8] that introduces a routine to reduce anxiety in children with ASD. First, there is a session start time that involves going around the table to talk about the week, a reminder about the previous session, and a presentation of the day’s session with the suggested activities. Next, comes the time for working on social skills (didactic teaching and then training in the explicit group; cooperative games and then a summary and discussion in the implicit group). The third part is the end of the session, with a wrap-up of the session, possibly a snack, or another routine.

There are two types of social skills training groups, depending on whether implicit or explicit learning is preferred.

Explicit groups are structured around a specific program with progressive themes by session [8] and may follow a manual with different modules. This approach is a top-down modality consisting of theoretical teaching, often accompanied by a summary sheet, that is then put into practice with repeated exercises, scenarios, role-playing to attempt to automate social rules, and/or exercises to complete at home [8,39,52]. The criticism raised by several authors pertains to the essentially didactic approach of certain explicit groups. Some children with ASD who participate in these groups have been able to learn problem-solving strategies but are not able to apply them in a situation [53], which is consistent with the “natural hypersystemizing” that Baron Cohen described in this population [54]. Therefore, a generalization problem seems to exist [39,50,52,55].

In implicit groups, social skills come from an ecological situation through a bottom-up modality, with the objective being to allow the subject to learn the social skills on their own and, secondarily, exposing him or her to social situations. Children with ASD experience difficulties through these group situations, and they may look for solutions and/or receive assistance from a group leader or even other children. Second, a summary is performed by the leader with the children. These groups most often use play as a medium for greater motivation and to facilitate cooperation among the children [55]. For example, games such as the Social Skills Game, Social Skills, and the SociaBillyQuizz are utilized in France [55,56]. However, cooperative implicit groups can be formed by creating social situations in a group or by transforming games into cooperative games. There is no theoretical learning but rather immersion through a game, followed by group discussions to overcome any problems that were encountered and identify solutions together. With this type of implicit learning, generalization would be beneficial [49,55]. This benefit is what led Jonsson and Williams to advocate for the development of groups with a bottom-up approach, meaning that they used implicit learning [49,57].

For our pilot study, we have focused on a social skills group that uses an implicit modality for children aged 9 to 11 years old with ASD.

The originality of this group involves its use of play, namely, through board games or individual games that we have transformed into collaborative games to develop cooperation among peers.

## 2) Study of the impact of an implicit social skills training group

### 2.1. Objective

Our primary objective was to assess the efficacy of social skills training groups in terms of socialization in children with ASD using an implicit modality involving a collaborative game.

### 2.2. Method

#### 2.2.1. Plan and population

- Design of the study: During the 2015/2016 school year, we conducted a before/after intervention study in a child psychiatry day hospital in Marseille, France involving participation in a social skills group.
- Inclusion criteria: We enrolled all children from 9 to 11 years old with ASD without ID who were treated for at least half a day each week in our unit and were involved in an implicit social skills group for 22 weeks. The ASD diagnosis was established clinically and was confirmed through standard observation of the child (Autism Diagnostic Observation Schedule, ADOS) [58]. The absence of an intellectual disability was verified, with a total IQ > 70 using the WISC IV [59]. The date range for participant recruitment was from 10/05/2015 to 10/09/2015, and the children were followed for 22 weeks, i.e., until 05/20/2016, excluding school holidays.
- Non-inclusion criteria: We did not enroll subjects who were participating in a specific social skills group (e.g., language pragmatics) or were using different methods.
- Exclusion criteria: We excluded children who had stopped participation.

We analyzed the data of all ASD children from 9 to 11 years old who were treated in our day hospital and were participating in our implicit social skills group without sampling because of the small population size.

#### 2.2.2. Conduct of the groups

The goal of the groups was to work on different social and emotional situations to develop mentalization, cooperation, and assertiveness abilities in children. To accommodate our population of young children, in whom it is important to maintain motivation, we adapted the sessions from week to week based on the needs of the children, their difficulties, and their expectations. We used play, including strategy games, board games, and individual games that we transformed into cooperative games by modifying the rules such that the only way for the children to finish the game was to communicate and cooperate, so they listened to each other and made an effort to understand one another. These groups made it possible to practice conversation skills, theory of mind, cognitive flexibility, and the exchange and management of emotions. Each of the weekly 30-minute sessions was structured in the same way, with routines for better child adherence, and was conducted by two neuropsychologists and a psychiatrist.

#### 2.2.3. Hypothesis

We hypothesized that the social skills of children with ASD would improve after participating in the implicit social skills group. If empathy improved after the intervention, we hypothesized that this improvement would primarily involve the cognitive dimension of empathy because social skills groups rely on strategies from cognitive and behavioral interventions [8,56].

#### 2.2.4. Judgment criteria

The children’s social skills were assessed before and after 22 weeks of group participation. Given the complexity of the social skills, their multidimensional character [12,49], and the lack of a baseline tool in French, we used a judgment criterion combining the Social-Emotional Profile (SEP), the Childhood Autism Rating Scale (CARS), and the EQ. The questionnaires for determining the EQ were completed by the parents, and the SEP and CARS were completed by the caregivers.

The SEP [60], a French adaptation of the Social Competence and Behavior Evaluation [61], is a standardized instrument for the heteroassessment of social skills and adjustment difficulties in children. It includes 80 statements that the evaluator scores on a Likert-type scale, which are aggregated into eight basic scales and four global scales. The first three basic scales (depressive—joyful; anxious—secure; angry—tolerant) describe the child’s emotional adaptation; the next three (isolated—integrated; aggressive—calm; egotistical—prosocial) describe his or her social interactions with the other children; and the last two (oppositional—cooperative, dependent—autonomous) describe the child’s social interactions with adults. Regarding the four global scales, the first refers to “Social Skills” and aggregates 40 exploratory positive statements, including “emotional maturity, flexibility, and positive adaptation in relationships with peers and adults;” the second and third scales assess “Internalizing Problems” and “Externalizing Problems.” Finally, the “General Adaptation” scale, which aggregates the 80 statements, provides the child’s overall adaptation level. A higher score in an explored area indicates a more favorable adaptation by the child in that area. The SEP measures social competence, helps distinguish emotional issues from behavioral issues and makes it possible to measure the effects of an intervention through repetition. For our survey, we were interested in the basic scales and the global “Social Skills” and “General Adaptation” scales, with the hypothesis that social competence and adaptation would improve after the children’s participation in social skills training groups [60,61].

The CARS is a heteroassessment scale for infantile autism [62] that was translated into and approved for use in French in 1989 [63]. It is a tool used to confirm an ASD diagnosis and is used as a disorder intensity scale to measure changes in the symptoms of autistic children with treatment. It consists of 15 items, each ranked from 1 to 4 points, with a maximum score of 60 points. The ASD diagnosis is defined as a score of 30 points or higher. A higher score indicates more intense symptoms. We were interested in the following four subdomains even more than the overall score: social relationships, emotional responses, adapting to change, and fears and anxiety. We hypothesize that the proposed social skills groups could improve these dimensions.

To assess the empathy level, which is considered a social skill [39,50,64], we used the EQ [30] for children and adolescents [31], which has been translated into and approved for the French language [65]. It is a multipart questionnaire that is completed by the parents to measure the empathy skills of their child through 40 statements describing everyday life experiences that utilize empathy skills [31]. The maximum score is 80 points, with a cut-off at 30, below which 80% of children with ASD without ID fall [65]. We were interested in the variation in the empathy quotient. Because the empathy quotient has good internal consistency, it is designed to remain stable without intervention [31]. We wanted to know whether the children’s empathy quotient changed after participation in the social skills training group. We then separated the items in the French-language questionnaire [65] depending on whether they referred more to cognitive empathy or emotional empathy, which are two components of empathy [30], to determine the most changed component in the event of a change in empathy. Thus, items 1, 2, 4, 5, 6, 8, 9, 11, 12, 13, 14, 15, 16, 18, 21, 22, 26, 29, 30, 31, 32, 34, 35, 36, 37, 38, and 40 referred to cognitive empathy. Items 3, 7, 10, 17, 19, 20, 23, 24, 25, 27, 28, 33, and 39 pertained to emotional empathy.

#### 2.2.5. Measurements

The SEP and CARS scores were recorded before the children participated in the social skills group. The children were hospitalized in our child psychiatry day hospital for several weeks before the first assessment. The scales were completed after observation of the children and gathering pertinent information from school by a two-person team (a neuropsychologist and a child psychiatrist who knew the child and had participated in the group) in a child psychiatry department in Marseille using a “consensus” method. This team retested the children with the assessment after the end of the group session by observing the children’s interactions and examining the information from teachers in school.

The empathy quotient questionnaires were completed by the children’s parents before participating in the group and then after 22 weeks of treatment.

The efficacy of the social skills training groups was evaluated to show an increase in EQ and SEP scores and a decrease in CARS scores.

#### 2.2.6 Data entry

An Excel file was created to collect before- and after-intervention data.

The children’s data were anonymized and identified only using a digital code, including the inclusion rank and the first letter of their name and surname (for example, 01EJ).

The data were checked by someone apart from the person who input the data.

#### 2.2.7. Statistical methods

For each child, we calculated the score difference for each scale and subscale (EQ, SEP, and CARS) between the two assessments, namely, before establishing the group and then after 22 weeks of social skills training. We averaged the differences and found the median. We then tested the hypothesis of an improvement in the scores after intervention using the Wilcoxon signed-rank test. The effect sizes were calculated using Rosenthal’s formula (r = Z/√n).

#### 2.2.8 Ethics committee

The protocol was submitted to the University of Aix-Marseilles Ethics Committee, which has granted its approval.

#### 2.2.9. Registration in ISRCTN registry

The study is listed on the ISRCTN registry with trial ID ISRCTN16636069. We registered this study only after the participation of autistic children in the social skills group because this study is considered in France to be a study about “routine care”—namely, a study concerning care usually proposed without invasive intervention—which does not require this registration.

The authors confirm that all ongoing and related trials for this intervention are registered.

### 2.3. Results

#### 2.3.1. Description of the population

**Fig 1:**
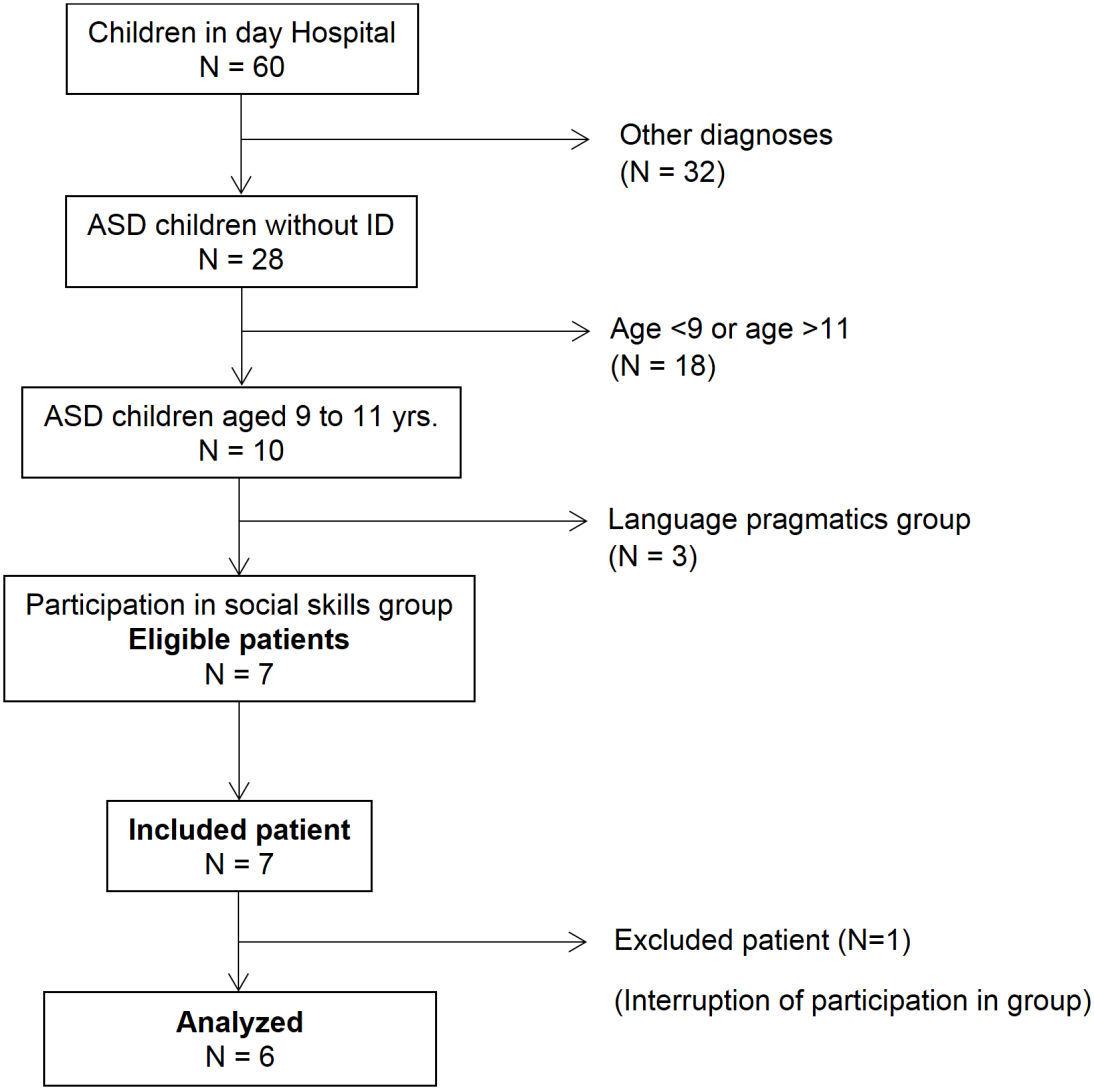
Flow chart.

We recruited a population of 7 patients. One patient was excluded because he left the group to participate in a sports activity at the same time. Our study population therefore consisted of 6 patients (Fig 1), with an average age of 9 years and 9 months (median age of 9 years and 8 months) and with 4 boys and 2 girls. All participants were enrolled in an ordinary (non-specialized) school in fourth or fifth grade and had a total IQ > 70. We present the breakdown of the WISC scores and specific ADOS-2 scores. The median initial SEP, CARS and EQ scores were 37.5, 31.75, and 8, respectively (average respective scores of 38.3, 33.16, and 8) (Table 1). The children in our group had never taken any medication previously; they did not receive medication during group period. All children had received individual psychotherapy treatment the year before participation in the group, and they also received this treatment during the group session with a psychologist or a resident in child psychiatry. Additionally, they received school lessons with 3 or 4 other children given by a specialized teacher (in addition to mainstream schooling) on the day of hospitalization.

**Table 1:** Baseline characteristics of the population.

		Patient n°						Mean	Median
		1	2	3	4	5	6		
Sex		M	M	M	F	F	M		
Age		9 yr5 mo	9 yr5 mo	9 yr8 mo	9 yr8 mo	10 yr3 mo	9 yr11 mo	9 yr8 mo	9 yr8 mo
WISC IV	VCI	69	120	82	101	90	81	90.5	86
	PRI	99	94	96	81	77	92	89.8	93
	WMI	73	109	91	70	85	88	86.0	87
	PSI	86	100	86	64	64	96	82.7	86
	IQ	75	109	85	74	73	85	83.5	80
ADOS2	SA	11	6	11	10	7	6	8.5	9
	RRB	1	1	1	1	1	2	1.2	1
	Total	12	7	12	11	8	8	9.7	10
	CS	7	4	7	7	5	5	5.8	6
SEP	General T1 adaptation	45	37	40	36	33	39	38.3	37.5
CARS	Total T1	33	30.5	38.5	30	29.5	37.5	33.2	31.8
EQ	Total T1	2	7	13	7	10	9	8.0	8.0

WISCIV: VCI: Verbal Comprehension Index; PRI: Perceptual Reasoning Index; WMI: Working Memory Index; PSI: Processing Speed Index; IQ: Intelligence Quotient

ADOS 2: SA: Social Affect; RRB: Restricted and Repetitive Behavior; CS: comparison score

SEP: Social-Emotional Profile; CARS: Childhood Autism Rating Scale; EQ: Empathy Quotient

#### 2.3.2. Principal results

**Fig 2:**
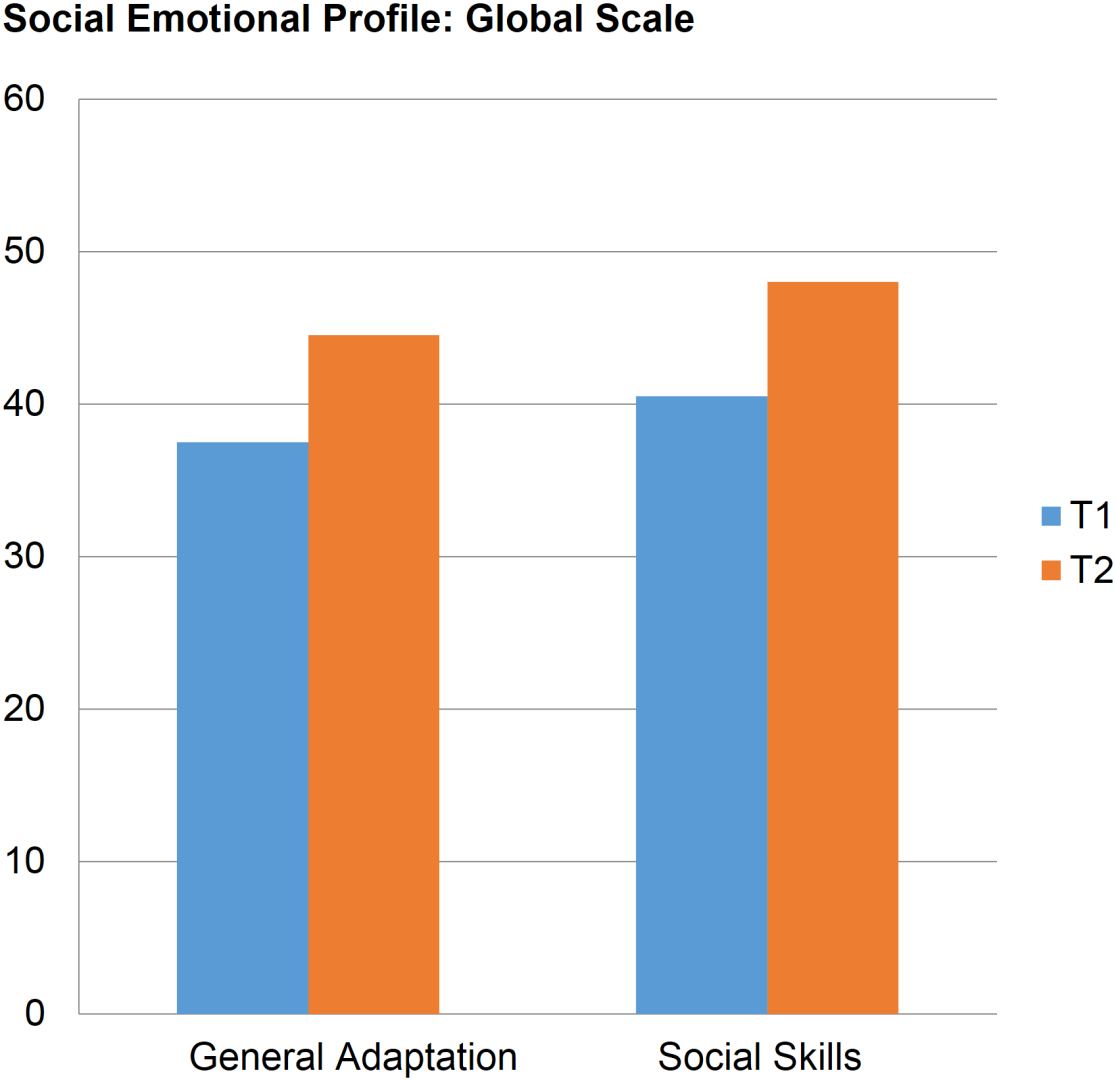
Median SEP results before (T1) and after (T2) the group.

In the SEP, there was a significant increase (p < 0.05, Wilcoxon test) of 8 points (mean: 7.7 points; min: 5; max: 11) in the median general adaptation T-scores and 7.7 points (mean: 6 points; min: 5; max: 8) in the social skills score after intervention. Six of the basic scales showed a significant change after 22 weeks of intervention. First, the egotistical-prosocial scale showed a T-score increase of 6.5 points, and the anxious-secure scale showed a median increase of 6 points. Also showing increases were the aggressive-calm scale (median increase: 5.5), the oppositional-cooperative scale (median: 4.5), the angry-tolerant scale (median: 4), and the isolated-integrated scale (median: 3.5). We did not observe any change in the depressive-joyful or dependent-autonomous scales (Table 2, Fig 2).

**Table 2:** SEP, CARS, and empathy quotient results before (T1) and after (T2) 22 weeks for an implicit social skills group (D = Score T2 –Score T1).

			Patient no.						Mean	Median	P value	ES
			1	2	3	4	5	6				
**SEP**	**General adaptation**	T1	45	37	40	36	33	39	38.3	37.5		
		T2	50	46	47	42	42	49	46.0	44.5		
		**D**	**5**	**9**	**7**	**6**	**9**	**10**	**7.7**	**8.0**	**0.028**	**-0.90**
	**Social skills**	T1	42	45	40	35	37	44	40.5	40.5		
		T2	50	52	47	40	39	51	46.5	48.0		
		**D**	**8**	**7**	**7**	**5**	**2**	**7**	**6.0**	**7.5**	**0.026**	**-0.91**
**CARS**	**Total**	T1	33	30.5	38.5	30	29.5	37.5	33.2	31.8		
		T2	30.5	27.5	35.5	24	24.5	32.5	29.1	29.0		
		**D**	**2.5**	**3**	**3**	**6**	**5**	**5**	**4.1**	**4.0**	**0.031**	**-0.90**
	**Social relationships**	T1	2.5	2	3	2	2.5	2.5	2.4	2.5		
		T2	2	1.5	2	1.5	2	2	1.8	2.0		
		**D**	**0.5**	**0.5**	**1**	**0.5**	**0.5**	**0.5**	**0.6**	**0.5**	**0.031**	**-0.95**
	**Adaptation to changes**	T1	2.5	2.5	3.5	2	3	2.5	2.7	2.5		
		T2	2	2	2	1.5	2	2	1.9	2.0		
		**D**	**0.5**	**0.5**	**1.5**	**0.5**	**1**	**0.5**	**0.8**	**0.5**	**0.031**	**-0.92**
**EQ**	**Total**	T1	2	7	13	7	10	9	8.0	8.0		
		T2	8	15	29	18	22	20	18.7	19.0		
		**D**	**6**	**8**	**16**	**11**	**12**	**11**	**10.7**	**11.0**	**0.027**	**-0.90**
	**Cognitive Empathy**	T1	0	2	6	2	0	4	2.3	2.0		
		T2	4	9	20	11	11	12	11.2	11.0		
		**D**	**4**	**7**	**14**	**9**	**11**	**8**	**8.8**	**8.5**	**0.028**	**-0.90**
	**Emotional Empathy**	T1	2	5	7	0	10	5	4.8	5.0		
		T2	4	6	9	7	11	8	7.5	7.5		
		**D**	**2**	**1**	**2**	**7**	**1**	**3**	**2.7**	**2.0**	**0.027**	**-0.90**

Regarding the CARS, we observed a significant reduction of 4 points (mean and median) in the total score after 22 weeks of treatment. This same trend was noted in the “social relationships” (median reduction of 0.5) and “adapting to change” (median reduction of 0.5) subdomains. There was no significant change in emotional responses and anxiety (Table 2).

**Fig 3:**
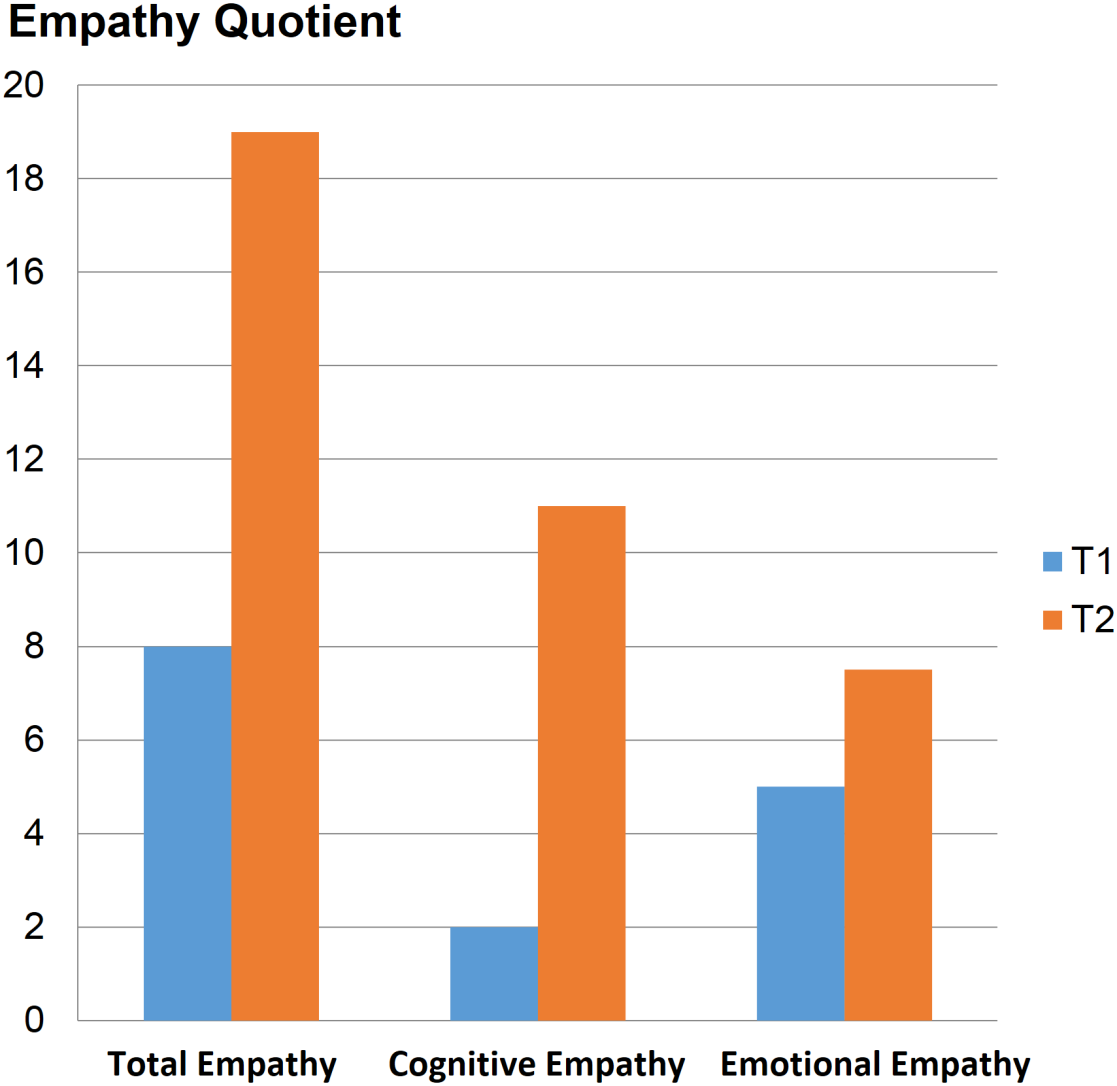
Median EQ results before (T1) and after (T2) the group.

The EQ increased two-fold after our intervention from 8 points to nearly 19 points. Empathy involving cognitive processes increased 5-fold, while emotional empathy was increased 1.5-fold (Table 2, Fig 3).

The effect sizes for SEP, CARS and EQ were greater than or equal to 0.9.

### 2.4. Discussion

#### 2.4.1. Discussion concerning the study population

Our population of ASD children was homogeneous in terms of age and level of schooling. Their IQ levels were superior to 70, confirming that the children with ASD in our group did not suffer from intellectual disability. The breakdowns of WISC scores showed a discrepancy between IVC and PRI but without a homogeneous tendency; IVC was superior to PRI for three children, and PRI was superior to IVC for the other three. Among the ADOS-2 algorithm, 3 children were in the autism spectrum (overall score superior to cut-off 7), and 3 were autistic (score superior to cut-off 9). Our group was fairly homogenous regarding the comparison scores of ADOS-2, which indicated mild or moderate autism. The baseline SEP scores placed our population at low general adaptation. The median CARS score was greater than 30 yet showed a mild to moderate level of autism in our population with different levels of severity. The empathy quotient was consistently low in the population, far below the threshold of 30—below which the majority of children with ASD fall—which was in line with the literature [31,65] and indicated low empathy levels in our population.

#### 2.4.2. Discussion of the primary results

In the SEP, there was an improvement in general adaptation, particularly in social skills, which is where we especially wanted to focus with the social skills training group. The improved basic scales T-score showed that after the intervention, the children were more adaptable to change, had more self-confidence (anxious/secure scale), and opened up more to others through the development of prosocial attitudes (egotistical-prosocial scale). They demonstrated more patience and tolerance and less excitement (angry-tolerant scale). They participated more easily in group activities, were more responsive to requests from other children, and better identified requests (isolated-integrated scale). The subjects cooperated more and were more accepting of others in their games (oppositional-cooperative scale). They engaged in conflict less and identified different response strategies (aggressive-calm scale). In the scales for which we observed significant improvement, many items involved consideration of the other person’s point of view, or theory of mind, with which autistic children have difficulty. Cognitive processes were therefore mobilized and appeared to improve after 22 weeks of treatment. Conversely, the scales for which there was no significant improvement (depressive-joyful and dependent-autonomous) included items involving emotional aspects, such as the sharing of feelings, pleasure, and crying. Based on this scale, therefore the observed improvement appears to mainly involve cognitive processes.

In the CARS, the significant reduction in the total score meant a reduced severity of autism, related to improved social relationships and better adaptability to changes. The emotional responses were unchanged. These two points are consistent with what was observed on the SEP scale, suggesting yet again that the observed changes resulted from improved cognitive skills due to the establishment of a social skills training group.

There was a clear improvement in empathy, as previously observed by Fritsch et al. for adults with ASD [66]. However, in our study, the children all remained below the threshold of 30, below which the majority of children with ASD without ID fall [31,65]. Thus, even though there was an improvement in empathy, difficulties still existed and will require continued treatment. Cognitive empathy improved more after our intervention than emotional empathy. Baron Cohen indicated that empathy is altered in different disorders, especially in ASD and psychopathic personalities. Unlike in psychopathy, cognitive empathy would be altered in ASD [32], which means that it is targeted in rehabilitation—with significant room for improvement—with social skills training groups.

The effect sizes were large, indicating a real change in social skills.

The results of the different scales were consistent: there was an overall improvement in social skills after 22 weeks of implicit social skills group training. This improvement was not limited to the group because the participants were observed in different environments by different observers. This improvement appears to involve the development of certain cognitive skills, such as theory of mind. This improvement is not surprising because social skills training groups rely on principles from cognitive interventions [8,47].

#### 2.4.3. Discussion concerning the measurement instruments

We wanted to use different scales because social skills are multidimensional, which makes assessment difficult when using a single instrument [49]. In addition, there is no reference tool [67], or rather, the tools are not suitable for ASD subjects [52]. Some authors, including us, have suggested assessing social skills by combining multiple tools [50,52,68]. Different instruments have been used in the literature [11,12,49]. In France, some have used the Vineland (VABS in French) or even the ADOS, which was designed specifically for autism [50]. However, we feel that these scales are too general for finely assessing social skills. Others have used the faux pas recognition test, the SRS [55], or even emotion recognition [40]. However, the SRS has not been approved in French, and the faux pas scale essentially assesses the theory of mind. Similarly, the facial emotion recognition scale, referenced by the HAS [6], evaluates only one aspect of social skills, resulting in learning but not standard social skills.

We chose the SEP because the scale focuses specifically on social skills and their use in daily life [60]. We wanted to include two other scales with the SEP. We added the CARS because it is commonly used in autism and thus provides an overall view of someone with ASD, in addition to assessing their social skills for a specific item. We then added the EQ because empathy is an altered social skill in autism [29,30,32,39,50,64] and because the scale is completed by the parents. We wanted to consider different environments and take different points of view into account to assess the change in the children’s social skills as objectively as possible after our training group. The SEP and CARS were completed by two-person caregiver teams. The EQ was completed by the parents based on what they observed in their daily life in another environment, which provided a good idea of the generalization of the acquired skills. Other authors have combined two scales to compare the points of view of teachers and parents [52,69,70]. The results of the three scales in our survey were consistent, giving more strength to our study because there was an improvement—without evaluator bias—in social skills, which appear generalized in daily life in an ecological situation.

If Reichow noticed an overall improvement in social competence with social skills groups [11], J. Gates showed only a medium overall effect in another meta-analysis that studied different informants (teachers, parents, observers and youths). The effect was not significant for teachers and was small for parents and observers, which is why it is important to study the different environments in which children interact if we want to measure the effect of a social skills group [71].

Indeed, the generalization of social skills acquired in a group setting is a limitation that is regularly cited in the literature [11,12,39,49,50], generally for explicit groups, because it is then difficult for the subject to appropriate the proposed strategies [55] and apply them to daily life [50]. The advantage of implicit groups is that learning is done based on ecological conditions, so acquired skills can more easily be generalized to daily life. In implicit groups, play has a special place. It can ease inhibitions generated by the group situation and boost social skills through play [55] through a top-down modality. The SociaBillyQuizz is the first example [55]. The authors built a board game with cards on social skills with which children suffering from Asperger syndrome played during group sessions. The settings of the group, particularly the small size of population and the chronological structure with different parts (welcome, game, and conclusion), were the same as in our study. The authors explained that games are a good means of developing social skills and overcoming inhibitions associated with group situations. Moreover, play and board games allow for better generalization [55].

Beaumont also used a game in a social skills group for Asperger-affected children. This group relied on blended implicit and explicit learning. The authors created special software, “Junior Detective Computer Game.” They explained that they used a computer game because computers are a highly effective teaching medium for children with ASD. In this game, the children with ASD had to be “detectives” and decode the suspects’ thoughts and feelings. For this game, they benefited from virtual theoretical learning (explicit learning) and learning by exposure to virtual social situations (implicit learning). Next, they participated in a real social group to facilitate generalization. This group improved the social skills of the children [70].

The specific nature of our group was that it was exclusively implicit, using games that had been transformed into cooperative games, which should allow for even better generalization. In effect, the children could not complete the game if they did not solicit the help of their peers, which involved thinking about others, learning how to formulate a question, understanding what the other person says, and answering in an appropriate manner.

Another research team implemented an implicit social skills group that did not involve a board game but an “outdoor adventure program.” In this controlled study, young children with ASD in the intervention group had to complete challenging physical activities that “require cooperation and communication with peers and instructors.” There was significant improvement in social communication, social cognition, social motivation and autistic mannerisms [72].

In addition to the groups, another intervention methodology to improve social skills in children with ASD with implicit procedures is the “peer-mediated intervention.” These interventions typically occur in school settings with schoolmates [73] through games [74] or other activities with neurotypical peers. For instance, Corbett showed efficacy on social competence of a theater-based intervention with young, trained peer actors [75]. Barber demonstrated efficacy of the Stay Play Talk procedure: a neurotypical child was paired with a child with ASD and was taught to stay, play and talk with him during 20-min weekly sessions about communication skills [76]. This methodology appears to allow good generalization [73,75].

#### 2.4.4. Limitations

The main limitation of our study was the small size of our population. This point is itself related to how social skills groups work because they must be homogeneous and consist of a limited number of patients to be effective [8,38,39]. Thus, nearly 40% of studies on social skills groups involve fewer than 10 patients [12]. Despite the small size, we found significant results that demonstrated the efficacy of our group in acquiring social skills.

The second limitation pertained to the before/after design of the study. With this type of study, it is possible to observe significant differences before and after the intervention, but we cannot state that the observed difference is due exclusively to the intervention [77]. There may indeed be confounding factors. To overcome these confounding factors, it is preferable to use controlled studies with a matched control group. We did not want to use a control group of children with ASD who would not be offered treatment through social skills groups because it would be unacceptable to us to deprive the affected children of treatment that could help them. Most likely due to these difficulties, few controlled studies are available in the literature [11,49]. In our study, social skills were greatly improved on three independent scales completed by different evaluators, which helps reduce the risk of confusion bias.

Lastly, three of the studied children with ASD left our child psychiatry day hospital after the year of care. Hence, it was not possible to study social skills in a sustained manner over time. We expect that implicit learning with play and cooperative games in our group would have resulted in sustained improvement in social skills, as observed in a study by Pourre et al with an implicit social skills group. They observed a continuity of therapeutic benefits 6 months after the group ended [55].

## Conclusion

We have shown that an implicit social skills training group using cooperative games improved the social skills of children with ASD without ID. This improvement primarily affected their cognitive processes.

Implicit groups could allow better generalization of acquired skills than explicit groups. The results should be verified in a specific study to assess the long-term maintenance of the acquired skills. Comparison with an explicit group would also be interesting because it would show the strengths and weaknesses of these groups and their respective interest in the target population.

It would be interesting to combine implicit social skills group and peer-mediated interventions, which both are based on implicit procedures.
